# Altered Auditory BOLD Response to Conspecific Birdsong in Zebra Finches with Stuttered Syllables

**DOI:** 10.1371/journal.pone.0014415

**Published:** 2010-12-23

**Authors:** Henning U. Voss, Delanthi Salgado-Commissariat, Santosh A. Helekar

**Affiliations:** 1 Department of Radiology and Citigroup Biomedical Imaging Center, Weill Cornell Medical College, New York, New York, United States of America; 2 Speech and Language Center, Department of Neurology, The Methodist Hospital Research Institute, Houston, Texas, United States of America; 3 Department of Neuroscience, Baylor College of Medicine, Houston, Texas, United States of America; University of Lethbridge, Canada

## Abstract

How well a songbird learns a song appears to depend on the formation of a robust auditory template of its tutor's song. Using functional magnetic resonance neuroimaging we examine auditory responses in two groups of zebra finches that differ in the type of song they sing after being tutored by birds producing stuttering-like syllable repetitions in their songs. We find that birds that learn to produce the stuttered syntax show attenuated blood oxygenation level-dependent (BOLD) responses to tutor's song, and more pronounced responses to conspecific song primarily in the auditory area field L of the avian forebrain, when compared to birds that produce normal song. These findings are consistent with the presence of a sensory song template critical for song learning in auditory areas of the zebra finch forebrain. In addition, they suggest a relationship between an altered response related to familiarity and/or saliency of song stimuli and the production of variant songs with stuttered syllables.

## Introduction

Our ability to communicate vocally with one another is shared by songbirds at the level of sensorimotor vocal learning from tutors during development [1]. Studies in songbirds have revealed the generative mechanisms of vocal syntax [2], [3], [4], [5], [6], [7]. Our experiments have shown that zebra finches have a variable preference to learning aberrant or variant song syntax consisting of stuttering-like repetitions of song syllables [4].

**Figure 1:**
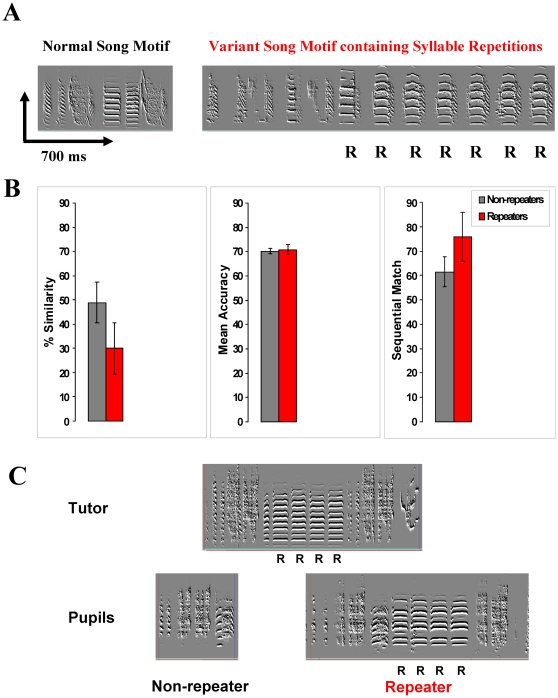
Song learning in syllable repeaters and non-repeaters. **A.** Spectrograms of non-repeater (left) and repeater (right) song motifs. The R's indicate repeated syllables. **B.** Learning of tutor's songs by tutored repeaters and non-repeaters. Bar graphs show no significant difference in % similarity, mean accuracy and sequential match of pupil song motifs with the respective tutor motifs, between repeaters (n = 8) and non-repeaters (n = 8) tutored by repeater tutors in this study. The error bars represent standard errors of the mean (SEMs) **C.** Spectrograms of the songs of a tutor and its two pupils as adults, one of which is a repeater and the other a non-repeater. Repeated syllables in the tutor's as well as repeater pupil's song are indicated by the letter “R”.

A small fraction (∼7%) of laboratory-raised male zebra finches produces a variant birdsong containing a variable number (three or more) of successive repetitions of song syllables [4]. Further, 50–60% of normal juvenile zebra finches learn to produce these repetitions when tutored by an adult repeater. However, the remaining 40–50% resist producing them despite such tutoring [4]. Figure 1A shows a comparison of the spectrogram of a normal song consisting of a stereotyped sequence of distinctive sounds with that of a variant song containing syllable repetitions. The mechanism underlying acquisition and production of syllable repetitions is unknown, and might be based on abnormalities in sensory or motor processes, or a combination thereof.

Sensory processing of song has been studied by electrophysiological recordings and molecular immediate early gene (IEG) product expression methods. Auditory responses have been recorded globally [8] and in auditory areas, namely field L, caudal medial nidopallium (NCM) and caudal mesopallium (CM), as well as in the song nuclei HVC, lateral magnocellular nucleus of the anterior nidopallium (LMAN), area X of the medial striatum, and interfacial nucleus of the nidopallium (NIf) [9], [10], [11], [12], [13], [14], [15], [16]. Topography of sensory activity has been determined by examining the spatial patterns of song stimulation-induced upregulation of IEG products such as ZENK [17], [18], [19], [20]. Tonotopic organization of song syllables has been detected in the canary NCM with whistles of increasing frequency being distributed along the dorsoventral axis [21], [22].

Functional magnetic resonance imaging (fMRI) of the songbird brain during auditory stimulation shows a robust blood-oxygen-level-dependent (BOLD) response in field L, NCM and CM [23], [24]. Our fMRI experiments on awake zebra finches reveal a distinctive topography of BOLD responses to female-directed songs differing in familiarity and significance. fMRI of the live zebra finch brain allows us to estimate differences in the strength of sensory representation of songs in relation to their salience with respect to a bird's learning history and perceptual experience. In this paper we use the fMRI approach to test whether there is any alteration in sensory song representation in birds that produce variant songs containing multiple (three or more) successive repetitions of syllables. We find a significantly reduced BOLD response to the tutor's song, but an enhanced response to an unfamiliar conspecific song in these birds compared to non-repeater birds. Responses to a non-vocal pure tone and bird's own song remain unchanged.

## Methods

### Raising and training of zebra finches

Birds were raised in the Methodist Hospital Research Institute vivarium in accordance with a protocol approved by the Methodist Hospital Research Institute/Texas A & M Institute of Biotechnology institutional animal care and use committee (Assurance Number - A4555-01, Protocol Number - 05011-R2). The song tutoring and recording procedure was similar to that described in a previous paper [4]. The 16 male birds used in this study were song isolated as hatchlings from 7 through 20 days post-hatch in a separate room. They were tutored in groups of 2–4 by an adult male producing repeater song from post-hatch day ∼20 until post-hatch day ∼100. At this stage female-directed songs were recorded for 30 min at least three times in sound isolation boxes to ascertain that the bird sings a stereotyped crystallized song. After day ∼100 the birds were housed in groups of 4–8 in large wire cages containing only repeaters or non-repeaters in separate rooms. These birds were tutored by eight repeater tutors in all. They were healthy birds that were available to us at the time period over which the scanning experiment was done. Among them one pair of non-repeater pupils each came from three tutors with no pupils having become repeaters, one repeater pupil each came from two tutors with no pupils having become non-repeaters, one non-repeater pupil and one repeater pupil came from one tutor, and one non-repeater pupil and four repeater pupils came from one tutor. One repeater pupil learned its song from one of three repeater tutors in adjacent cages. The tutor was not the father of the pupils. None of the repeater pupils repeated the same syllable as their tutor. The repeater and non-repeater birds were treated the same way as far as tutoring, housing and testing are concerned, so it is unlikely that this factor contributes to the differences between them. Songs of repeaters were tested for repetitions at multiple times over periods of several weeks to months after completion of tutoring. They were exposed to non-repeaters only in the short periods before and after the scanning sessions. The repeaters used in this study showed persistence of songs with syllable repetitions at all times that they were tested.

Songs used for determining tutor-pupil similarity were recorded after the birds were older than 150 days post-hatch. The tutor-pupil song motif similarity was measured with the Sound Analysis Pro program (http://forum.sci.ccny.cuny.edu/Members/ofer/sound-analysis-pro/sound-analysis-pro-2) using the asymmetric as well as symmetric time course comparison methods [25], [26]. We chose the symmetric method for motif comparisons in addition to the standard asymmetric method because the former method would take into account the song syntax (i.e. syllable order) as well as the acoustic features of individual syllables. The asymmetric method on the other hand compares segments or chunks of song motifs to each other disregarding their order or sequence within the motif.

### Preparation of birds for scanning

At the time of scanning the birds were between ∼150 to ∼300 days old. They were sedated with 40 µl Diazepam (Abbott Labs; 1.66 mg/ml diazepam in normal saline solution I. M.) 10 min before MRI scanning, and immobilized in a restraining device made of soft transparent Tygon plastic tubes (Saint-Gobaine) and a solid plastic tube (Kendall). The solid tube served as the core of the MRI coil. The dose of diazepam used for sedation was ∼5 mg/kg body weight. We have determined that surface evoked potential responses to the stimuli are not significantly altered by diazepam at this dose [24]. This dose is less than that (7.5 mg/kg body weight) used to show an increased bird's own song (BOS) sensitive response in HVC by Cardin and Schmidt [27]. At this dose the bird is sluggish, and it perches on the floor rather than on the perch. The effect sets in within 5 min and lasts for ∼2 – ∼3 hours. The fMRI experiments lasted for less than 2 hours after diazepam injection. After the experiments, the birds appeared to be still sedated. To minimize the contribution of the sedation level to the observed effects, we pseudo-randomized the order of the stimulus application, or performed MRI scanning on more than one day. The change in sedation level over time is unlikely to be responsible for the effects because the timing of any given stimulus was not the same in each session and in each bird with respect to the time of injection of the sedative. Scanning on different days was necessitated by the fact that some of the sessions were contaminated by excessive head motion and eye movements in the awake bird, and therefore they had to be repeated on another day. In a few of these experiments we were also concerned about the fact that the effect of sedation might be wearing off over time in the long scanning sessions. The physical conditions between different sessions on the same day or on different days were not found to vary significantly on our scanner either in animal or in human fMRI experiments conducted by the first author over the last several years. Changes in temperature are not a concern in our experiments because our birds were awake mildly sedated, not deeply anesthetized as in other fMRI studies in songbirds. No significant response failure rate has been evident in any of our scanning sessions in the present study, as well as two published prior studies [24], [28]. The apparent lack of response in seven instances (see *Results*) is a subthreshold phenomenon caused by a conservative multiple test corrected thresholding, and does not mean that the experiment failed in these cases.

The restrained birds were placed in a foam/rubber compound sound isolation box. Auditory paradigms were delivered using a flash memory music player (Samsung), a headphone volume booster (Radioshack, Inc.), and a pair of stereo headphones (COBY CV-200) with the magnets removed. The two headphone parts were randomly exchanged between the right and left side. Their distance to the bird's head was 4 cm. Auditory stimuli were calibrated by obtaining a sound pressure level at the head position of ∼100 dB for a continuous pure tone stimulus (modified RadioShack sound level meter with remote sensor). The background noise during the EPI sequence was ∼83 dB. The experiment was approved by the Institutional Animal Use and Care Committees of the Weill Cornell Medical College and The Methodist Hospital Research Institute/Texas A & M Institute of Biotechnology.

### MRI data acquisition

The MRI scanning and auditory stimulation protocols used in this study are in line with those used in our previous studies [24], [28]. Images were acquired on a GE Signa Excite 3.0 T whole body scanner using a custom built solenoid transmit/receive coil (length: 20 mm, inner diameter: 15 mm). The fMRI imaging pulse sequence was a four-shot 2D gradient echo planar imaging (EPI) sequence with TR/TE  = 1000/25 ms. The effective repeat time per volume thus was 4 s. Eight sagittal slices of 1.0 mm thickness, 4 cm FOV (phase FOV = 0.75), flip angle 70° and a matrix size of 128×64 (zero filled to 128×128) were acquired with gradient ramp sampling. Slices were prescribed from right to left. The scan time per stimulation was 1024 s (256 repeats). Additionally, localizers, in-plane anatomical images, and field correction maps were acquired.

### Auditory stimulation paradigms

All auditory stimuli were delivered in 16 blocks each consisting of a 32 s “on” and a 32 s “off'” segment, amounting to 1024 s total. To prevent amplitude clipping and to adjust stimuli to each other and the 100 dB reference tone, stimuli where normalized with respect to peak amplitude. Stimuli were played out 16 times, twice per sampling interval, during the “on” segment of each block. This way, the number of stimuli per time interval was kept constant, and stimuli could be synchronized to the sampling times. The auditory stimuli consisted of: A pure tone of 2 kHz frequency (TONE), a conspecific song motif (CON), the bird's own song motif (BOS), and the tutor's song (TUT). BOS and TUT were dissimilar to CON (similarity <20% with asymmetric time course comparisons and <40% with symmetric time course comparisons, Sound Analysis Pro program [25], [26], the latter one being an unfamiliar song recorded from a bird in a different colony (from the laboratory of Ofer Tchernichovski at City College of New York). The same conspecific song was used in all birds. All songs used as stimuli were female-directed songs recorded from birds that were >150 days old. The duration of TONE was 1000 ms, the duration of CON was 730 ms, the mean duration of BOS was 967±202 ms (non-repeaters; mean ± standard deviation) and 1513±353 ms (repeaters), and the mean duration of TUT was 1587±382 ms (non-repeaters) and 1348±369 ms (repeaters).

### Data analysis

#### Preprocessing

BOLD sensitive fMRI images were corrected for distortions caused by magnetic susceptibility artifacts using field correction maps [29]. The images were de-spiked and motion corrected using AFNI [30]. Data were smoothed slice-wise with a 2D Gaussian filter (half width 1.5 voxels), voxel-wise linearly de-trended, and temporally smoothed by convolution with a binomial filter over three time points. For each bird and stimulus, statistical significance of activation was defined voxel-wise by the correlation coefficient of the signal amplitude with the “on-off” block stimulation function. In this process, the motion correction parameters were taken as nuisance parameters and were regressed out.

The statistical analysis of the BOLD response was performed in two ways: 1. Statistical parametric maps (SPMs) of the whole brain; 2. Region-of-interest (ROI) based analysis with a) two-way analysis of variance (ANOVA) tests and post-hoc scatter plots with nonparametric testing and b) one-way ANOVA tests on per-bird normalized data. Whereas the SPMs amount to a fixed-effects analysis, the ROI-based analysis amounts to a more conservative random effects analysis. These approaches are described in detail below:

#### 1. SPMs

From the averaged correlation coefficients corresponding t-scores were computed. The multiple test problem was taken into account by correcting the t-scores using Gaussian random field theory [31], [32]. The so obtained SPMs were registered to a brain template which consisted of the least distorted and most symmetric EPI scan with respect to the sagittal midline. The 2D registration was based on a locally affine, globally smooth transformation [33] estimated from the EPI data and applied to the SPMs and averaged BOLD response time series. These transformations (as applied to the EPI data themselves) were visually inspected. They yielded an excellent match between anatomical and EPI images for the two midline slices. For more lateral slices, which were not used for a statistical ROI analysis, the match was less perfect but still sufficient to produce meaningful SPMs. Activations in areas with an EPI signal baseline less than 20% of the maximum slice amplitude were discarded. Eye components were removed. Activations were finally displayed as color-coded SPMs, averaged over the two hemispheres. In these, the display threshold for bird- and hemispheric-averaged activations was set to p = 10^−6^ (multiple test corrected using Gaussian random field theory and taking the n = 8 birds per group into account).

#### 2. ROI-based statistic

**Figure 2:**
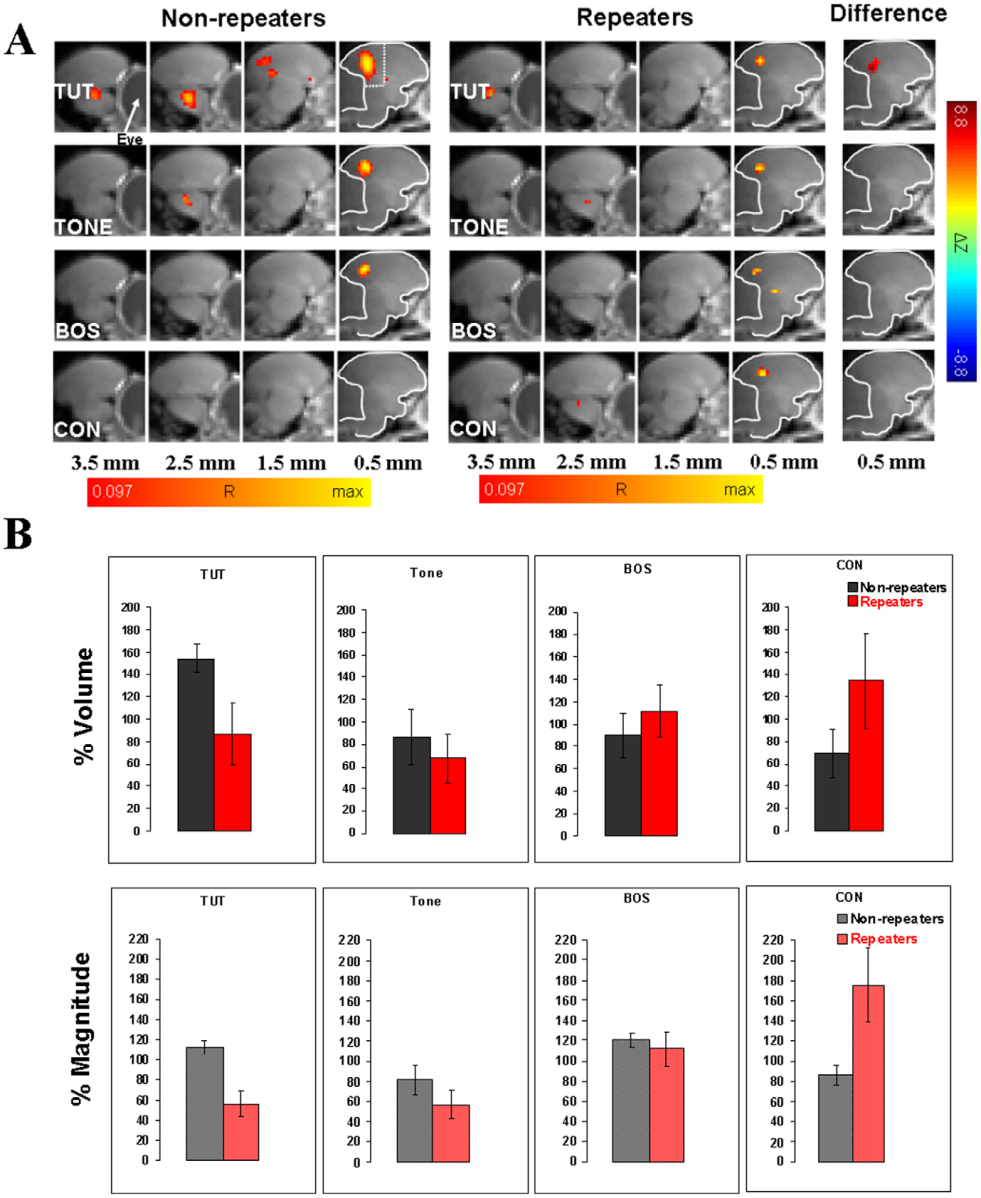
Functional MRI in syllable repeaters and non-repeaters. **A.** BOLD activations averaged over 8 non-repeater birds (“non-repeaters”) and 8 birds with syllable repetitions (“repeaters”) for the four auditory stimuli, tutor's song (TUT), 2 kHz pure tone (TONE), bird's own song (BOS) and conspecific song (CON). The type of stimulus is indicated on the left of each panel. The distance of the slice midlines from the midline of the brain is shown at the bottom. For the next-to-midline slice, the outline of the cerebrum is indicated, and in the first set of images the region of interest is shown as the area between the dotted lines and the border of the cerebrum. In the images labeled “Non-repeaters” and “Repeaters”, colors denote correlation coefficients R, which are overlaid on corresponding anatomical brain MRI sections. In the images labeled “Difference” color corresponds to the difference in z-values (non-repeaters – repeaters). The red end of the color scale corresponds to R = 0.097 or ΔZ = −8.8 (each corresponding to p<10^−6^, multiple test corrected). The yellow end of the R color scale is scaled individually. Activations from the right and left hemispheres of the brain are averaged. The main activated area that is consistently activated in all images corresponds to L/CM/NCM region. Even though CON activates the auditory areas in all non-repeater birds, unlike repeaters, no activated voxels are seen in the averaged images of non-repeaters. This is most likely because there is less consistency in non-repeaters compared to repeaters in the precise location of activated voxels. In accordance with this, the difference image does not show significant differences. **B.** Comparison of the volume and magnitude of BOLD response in the medial posterior area containing field L and small portions of CM and NCM for TUT, TONE, BOS and CON stimuli in repeaters and non-repeaters. Comparisons of normalized data between non-repeater and repeater birds for each stimulus, corresponding to the shown bar plots, using Student's t test show statistically significant difference for TUT with respect to both % magnitude (p = 0.003, df = 7) and % volume (p = 0.05, df = 7). For CON the difference is significant with respect to % magnitude (p = 0.048, df = 7), but not with respect to % volume. The corresponding values for TONE (% magnitude – p = 0.27, df = 7; % volume – p = 0.56, df = 7) and BOS (% magnitude – p = 0.64, df = 7; % volume – p = 0.48, df = 7) are not significant at the 5% error probability level. The error bars in the figure represent SEMs. A two-way ANOVA on the (non-normalized) BOLD response magnitude data over both groups and stimuli gives for the stimuli p = 0.02 (F = 3.52), for the groups p = 0.24 (F = 1.41), and for the interaction p = 0.01 (F = 3.84). The corresponding values on the BOLD activation volume are p = 0.20 (F = 1.61), p = 0.37 (F = 0.82), and p = 0.47 (F = 0.85), respectively. Two-way ANOVA only makes sense if the data are not normalized because the within-bird normalization erases differences with respect to groups. These results are overall consistent with the non-parametric analysis on the non-normalized data shown in Figure 3.

**Figure 3:**
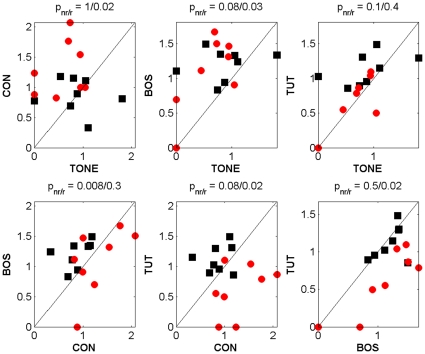
Pairwise comparisons of BOLD responses. Scatterplots of magnitude of BOLD response in the medial posterior forebrain, comparing the four different stimuli, namely TONE, conspecific song (CON), bird's own song (BOS) and tutor's song (TUT), pairwise in medial parasagittal slices, in 8 repeaters (red dots) and non-repeaters (black dots) each. Note that most of the red dots fall below the diagonal when TUT is compared with CON and BOS. p-values at the top of each panel indicate significance for the hypothesis that the two stimuli of that panel elicited the same response (Wilcoxon signed rank test), for non-repeaters and repeaters each.

Data pertaining to the volume and magnitude of BOLD activation consisted of the number of significantly active voxels and the mean amplitude of BOLD response calculated across all activated voxels, respectively, in an ROI defined on the template matched data. The ROI was defined as the whole posterior forebrain area across the two medial slices, extending 1 mm each to the right and left of the medial plane between the two hemispheres. Its rostral-caudal and dorsal-ventral extension was defined sufficiently large as to completely include the clusters of activation seen in the SPMs of the two medial slices. It is shown in Figure 2A in the panel corresponding to group “Non-repeaters” and “TUT” stimulation. Since the cerebellum almost completely drops out in EPI images, it does not contribute any signal exceeding the baseline threshold, and thus it was not necessary to manually outline the ROI with respect to its posterior border. The relative amplitude of the BOLD response was calculated for each voxel as the time-averaged relative change of the BOLD signal between the stimulus “on” and “off” phases, and expressed as percentages of the time-averaged signal. It was computed for all voxels that proved to be significant with respect to a voxel-wise (uncorrected) error probability of p = 0.005. This p-value would correspond to a multiple test corrected p≈0.05. (The group-averaged SPM's described before are based on a smaller p-value because of the larger amount of data averaged.) The same significance threshold was used to define the *volume* of the activation, i.e., the number of activated voxels in the ROI. Finally, the *magnitude* of activation was defined as the relative BOLD response amplitudes averaged over all significant voxels in the ROI. These measures of volume and magnitude were used in the results of Figure 3, which show individual responses for all birds. The p-values in Figure 3 at the top of each panel indicate significance for the hypothesis that the two stimuli of that panel elicited the same response (Wilcoxon signed rank test), for non-repeaters and repeaters each.

**Figure 4:**
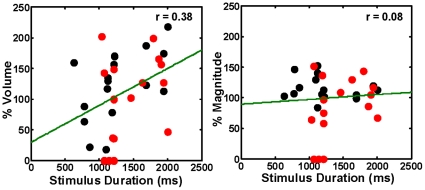
Absence of significant high correlation between the stimulus duration and BOLD response. Plots of % volume and % magnitude of the BOLD response as a function of the song stimulus duration. The stimuli represented here are those that showed variable duration, namely BOS and TUT. The BOLD response does not show a significant high degree of systematic correlated change with the duration of the stimulus in repeaters (red dots) and in non-repeaters (black dots). The green lines depict linear fit to the data.

**Figure 5:**
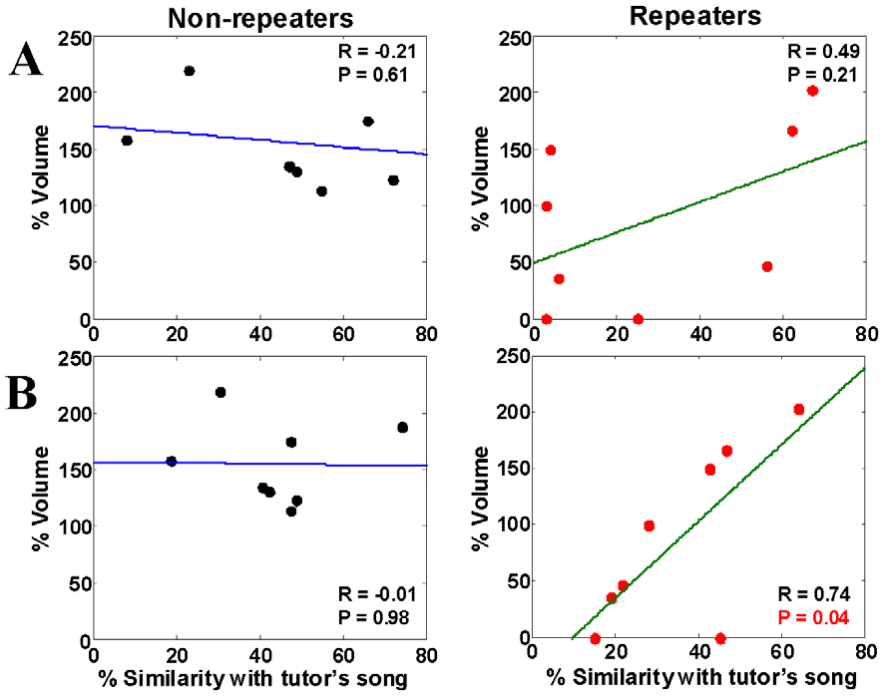
Correlation of BOLD response with tutor's song similarity. The volume of response to TUT in non-repeaters and repeaters, expressed as a percentage of the mean of all stimuli in each bird, is plotted as a function of the % similarity of the pupil's song with that of its tutor. The % similarity values obtained with the asymmetric time course motif comparison method (A) using the Sound Analysis Pro program [25], [26] show no statistically significant correlation in repeaters (red dots) or non-repeaters (black dots); however, values obtained using the symmetric method (B) shows positive linear correlation in repeaters, but not in non-repeaters. Linear fit to the data is indicated by the blue and green lines.

In the graphs of Figures 2B, 4, and 5, and all one-way ANOVA tests mentioned in the text, the following normalization procedure was performed to take into account differences in the overall responsiveness of individual birds: All ROI based values were normalized with respect to the mean across all four stimuli in each bird and expressed as “% volume” and “% magnitude” for the mean number of activated voxels and the mean amplitude of the BOLD response, respectively. A value of 100% corresponds to the mean value across the four stimuli. All raw BOLD values were measured as percent deviation from the baseline. The BOLD signal amplitude values vary substantially across birds. Therefore, within bird normalization, and expression of each value as a percentage of the mean response amplitude for all stimuli in each bird is a better quantity for comparison between birds here. In Figure 2B, the values for % volume and % magnitude of the BOLD signal were averaged over all birds in each of the two groups (non-repeaters and repeater birds). All computations were performed using in-house software written in MATLAB (The MathWorks), IDL (ITT Visual Information Solutions) and Excel 2003 (Microsoft Corp.), on PCs and Linux workstations.

#### Anatomical analysis

Areas of activation were localized by comparison with the location of field L and the cerebellum in the three-dimensional zebra finch MRI atlas kindly provided by C. Poirier [34]. We observed a good match of the cerebral/cerebellar outline in sagittal slices next to the midline between this atlas and our own high-resolution images. The cerebrum and cerebellum were manually outlined from an anatomical image of the zebra finch brain and overlaid to our SPM images.

## Results

To compare possible functional differences in sensory representation between normal singers (non-repeaters) and birds producing songs with more than two repetitions of a syllable (repeaters), we performed fMRI scans during auditory stimulation in 16 adult male zebra finches. Eight of them were repeaters and the other eight were non-repeaters. Their age ranged from 150 – 300 days. The birds were divided into repeaters and non-repeaters in accordance with criteria outlined previously [4]. All birds were raised with adult repeater tutors from post-hatch day ∼20 through day ∼100 in clutches of 2 – 4.

As far as song learning is concerned, as reported in a previous study [4], in these birds also there is no significant difference in the tutor-pupil song similarity measures of song learning between tutored repeaters and non-repeaters (Figure 1B). The mean % motif similarity (t = −1.39, p = 0.19 and df = 14), accuracy (t = 0.27, p = 0.79 and df = 14) and sequential match (t = 1.22, p = 0.24 and df = 14) in the two groups of birds were not significantly different. Thus both types of pupils imitate their tutor's song motif nearly equally well. On similarity and sequential match measures of song learning the non-repeaters were not significantly different from normally raised birds tutored by live normal tutors in the laboratory of our collaborator Ofer Tchernichovski (similarity t = 1.31, p = 0.21, df = 11; sequential match t = 0.56, p = 0.58, df = 11; unpublished data of Maul and Tchernichovski). There was a small (11%) but significant difference in the accuracy measure (t = 4.40, p = 0.001, df = 11), with respect to non-repeaters. Taken together these data suggest that the difference related to repetition of song syllables cannot simply be explained by poor learning. The repeated syllable in the songs of repeater pupils may be different in acoustic profile from that produced by their tutor. So the possibility that they are poor learners still exists. The repeaters appear to learn the tendency to repeat from their tutors without necessarily copying the repeated syllables from the tutor's song [4]. Figure 1C shows song motifs from two pupils, a repeater and a non-repeater, who learned their songs from the same tutor whose song motif is shown. It is apparent that both pupils have copied equally well syllable features from their tutor.

### Reduced BOLD response to tutor's song in syllable repeaters

During fMRI, birds were awake, restrained, and mildly sedated. Eight parasagittal images were obtained in each bird during stimulation with TONE, CON, BOS and TUT. All 16 birds showed significant and reproducible stimulus-evoked BOLD activations of widespread areas of the forebrain. Activations were bilateral. Both repeaters and non-repeaters showed significant stimulus-specific differences in BOLD response magnitude (for % magnitude in non-repeaters F = 3.69, p = 0.023; in repeaters F = 6.32, p = 0.002, one-way ANOVA). For the volume of activation the differences for non-repeaters, but not repeaters were significant (for % volume in non-repeaters F = 3.37, p = 0.032; in repeaters F = 0.96, p = 0.42, one-way ANOVA). However, closer examination reveals a difference between tutored repeaters and non-repeaters in fMRI activation of regions corresponding to auditory areas field L, and to a lesser and variable extent, CM and NCM. The BOLD response to tutor's song is significantly reduced in repeaters compared to non-repeaters in relation to CON and BOS in terms of both volume and magnitude (Figures 2 and 3). This difference appears to be stimulus specific. There is no such reduction in repeaters when responses to TONE and BOS are measured. Figure 2A shows that these results are strikingly evident in the medial brain slices in averaged data from all birds. Scatterplots of non-normalized BOLD response magnitude in Figure 3 highlight the weaker responses to TUT in repeaters by the fact that data points (red dots) from nearly every single repeater fall below the unity ratio diagonal when CON and BOS are plotted against TUT. Statistical significances for this difference in response are given at the top of each panel. An analysis of the volume of the BOLD response (not shown) reveals a similar trend: In non-repeater birds, there is a significantly larger volume of response to TUT than to CON (p = 0.04) and BOS (p = 0.03), whereas in repeater birds the response to TUT does not differ from CON and BOS (p = 1 for both cases). Most of the BOLD activity is in field L. However, similar to our previous observations [24] variable amounts of activity is seen extending into CM and NCM.

In terms of comparison with the aforementioned normally raised birds from the Tchernichovski laboratory, we find that there are no statistically significant differences in the magnitude and volume of the BOLD response to CON and TUT stimuli between their birds and our non-repeaters. Three out of four of these measures are quantitatively similar in the two sets of birds. (Data were obtained as part of a different experiment [28] and are not shown here.)

### Greater responsiveness to unfamiliar conspecific song in repeaters

The fact that decreased response to TUT in repeaters is unlikely to be due to a non-specific reduction in auditory responsiveness to sound or to songbird vocalizations, in general, is supported by an additional result. The BOLD response to a conspecific song that the bird has never heard before, is significantly stronger in repeaters compared to non-repeaters in its magnitude (Figure 2, p = 0.048, df = 7, Student's t test). This result, combined with the differential responses to TUT and BOS, suggests a complex interplay between processes that mediate the recognition of familiarity, saliency and ownership of the sounds that the bird hears, with regard to the altered song produced by the repeaters.

### No role for short term plasticity and variable stimulus duration in BOLD response changes in repeaters

Since stimuli were presented sequentially during each scanning experiment (wherever possible, all four stimuli were tested in one or two single sessions with three exceptions, in which one stimulus was added in one additional session), we considered the possibility that the order of stimulus presentation might be responsible for the response alterations observed in repeaters. Such an effect might be seen if repeated stimulation induces short term changes in response that cross over from the preceding stimulus to the succeeding one. Two lines of evidence in our experiments indicate that this explanation cannot account for the differences in responsiveness to TUT and CON between repeaters and non-repeaters. First, the stimuli were delivered in a pseudo-random order within one session or with a gap of several days or weeks between sessions, in cases where more than one session was necessary to conclude the experiment. We find for example that there is no significant difference between responses to TUT when it is presented at position 2 or position 3 in the stimulus presentation order (% magnitude in non-repeaters at position 2 – 117.6±12.5, n = 3; at position 3 – 117.7±2.1, n = 3, p = 0.99; % volume in non-repeaters at position 2 – 159.6±15.3, n = 3; at position 3 – 168.7±30.6, n = 3, p = 0.80, Student's t test). Second, if the stimulus order rather than the type of stimulus determined the magnitude of the response, as would be the case if short-term cross-stimulus habituation or potentiation were occurring, we would see a corresponding consistent declining or rising trend in the volume or magnitude of the BOLD response, as a function of stimulus order. We did not observe any such consistent trend in our birds (not shown).

The song stimuli used vary in duration, in part because song motifs containing repeated syllables tend to be longer than song motifs produced by non-repeater birds. We therefore asked whether the BOLD response varies systematically with the duration of the song stimulus by assessing the correlation between the two sets of values for BOS and TUT stimuli. We used these stimuli for this analysis because they are the only stimuli that varied in duration. Figure 4 shows that there is no high correlation of % magnitude or % volume of the response with stimulus duration in repeaters and in non-repeaters. The mean duration for TUT and BOS stimuli used in repeaters and non-repeaters are also not significantly different from each other: A two-way ANOVA test over the stimulus lengths and the two factors, non-repeater/repeater and BOS/TUT duration, gave F(15) = 0.80; p = 0.66 for the group and F(1) = 2.46; p = 0.14 for the stimulus. To further assess the contribution of variable stimulus duration, we also performed an analysis of covariance (ANCOVA) on repeater and non-repeater data with stimulus duration as covariate. We found that for TUT stimuli the magnitude of the BOLD response is significantly smaller in repeaters compared to non-repeaters [F(1,13) = 11.492, p = 0.00483]. The BOLD response volume evoked by TUT is not significantly different [F(1,13) = 3.45, p = 0.086] between the two groups. These data suggest that there might only be a partial contribution of stimulus duration, if any, to the measured difference in responses to TUT stimuli between repeaters and non-repeaters. Furthermore, since the same CON stimulus was used in all birds, the difference in response magnitude in that case cannot be explained by changes in stimulus duration.

### Relationship of BOLD responsiveness with tutor-pupil song similarity

One question that arises from our observation of alterations in BOLD response in birds that produce aberrant or variant song is whether there is a correlation between how well a bird imitates its tutor's song and the volume and magnitude of BOLD activation of the auditory areas by that song. We directly measured this correlation in repeaters and non-repeaters. Figure 5A shows that in repeaters there is a no significant linear correlation between the volume of activation in response to TUT stimulation and the % song motif similarity of the song of the pupil to that of the tutor (r = 0.49, p = 0.21, df = 14). There is also no such correlation in non-repeaters (r = −0.21, p = 0.61, df = 14). If we use the symmetric time course method of motif similarity in the comparison between TUT and pupil's BOS then the correlation in repeaters becomes statistically significant (r = 0.74, p = 0.037, df = 14), while non-repeaters show no correlation (r = −0.01, p = 0.98, df = 14) (Figure 5B). Since BOLD activation to TUT is much weaker in repeaters than in non-repeaters, it is likely that the lack of correlation in non-repeaters is due to saturation of the BOLD response to TUT in the latter birds. There was also no correlation of % tutor-pupil song motif similarity with the mean BOLD response magnitude (r = 0.11, p = 0.79) in repeaters and non-repeaters (r = −0.2, p = 0.64).

One caveat to our interpretation of decreased BOLD response to TUT and increased response to CON in repeaters compared to non-repeaters is the possibility that these results might be explained by a differential familiarity to songs with syllable repetitions and no repetitions, respectively, in the two sets of birds in the intervening period between completion of song learning and testing for auditory responses. This explanation seems unlikely however because when we scanned two repeaters, and presented them with a CON stimulus containing repetitions, the BOLD responses evoked in them to that stimulus were greater than the responses to BOS and TUT, just like those obtained with CON stimulus containing no repeated syllables.

## Discussion

Our experiments tested whether changes in sensory processing of song might be associated with the changes in motor output observed in zebra finches producing variant song syntax containing variable repetitions of song syllables. We studied sensory representation of song by using fMRI to image the BOLD response to various stimuli in awake mildly sedated birds. The response to TUT in repeaters is significantly weaker than in non-repeaters, as measured by the BOLD response magnitude and volume in the field L region. The weak response cannot be explained by a general attenuation of auditory responsiveness in these birds because the responses to TONE and BOS measured under the same conditions remain unchanged. Moreover, volume and magnitude of response to CON are significantly stronger in repeaters compared to non-repeaters. The changes in BOLD responses to TUT and CON are unlikely to be due to an interaction between activity-dependent response plasticity and any systematic difference in the order of stimulus presentation between repeaters and non-repeaters because no significant cross-stimulus response habituation or potentiation was detected in our birds. The stimuli were presented in a pseudo-random order, and the order was not the same in each bird. Therefore habituation or changes in arousal due to recovery from the sedative cannot account for our data.

The differences in BOLD responses to TUT and CON therefore are likely to be specific to some feature of those stimuli. However, none of the physical sound parameters measured with Sound Analysis Pro, namely duration, mean power, mean Wiener entropy, mean frequency modulation and mean pitch shows any significant consistency across birds in the correlation with the BOLD response volume or magnitude, irrespective of whether we look at repeaters or non-repeaters (data not shown). Therefore, our results cannot be explained simply by a difference in processing of specific acoustic features between repeaters and non-repeaters. As far as TUT is concerned, because of the similarity of its acoustic features with the corresponding BOS stimulus, the difference might be related to its sensory memory or recognition of its historical significance in the life of the bird. In terms of the underlying mechanisms, at least two broad possibilities merit examination. Birds that have a tendency to become repeaters might not be able to form a robust auditory template of the tutor's song in the early sensory phase of song learning. Alternatively, repeaters might lack the ability to maintain a lasting memory of the learned tutor's song template. Deciding whether one or both of these hypotheses are true would require a longitudinal study during development. However, the question whether the differential responsiveness of auditory areas in repeaters and non-repeaters is a consequence of the learning history and perceptual experience cannot be answered based on the results presented here.

Chronic multi-unit recordings and immediate early gene expression studies have led to the hypothesis that the sensory template or memory of the tutor's song may reside in the higher auditory area NCM (sensory template hypothesis). Phan et al. have shown that NCM neurons possess response selectivity for the tutor's song, and the strength of this selectivity as measured by a decline in the rate of habituation is correlated with the fidelity with which the tutor's song is imitated by the pupil [35]. Bolhuis and co-workers have demonstrated a high degree of correlation between the extent of imitation of the tutor's song and the level of expression of the immediate early genes ZENK and c-fos in response to stimulation with it [36], [37]. Our result showing a decrease in the BOLD response to tutor's song in birds that produce an aberrant or variant song predominantly in field L implicates the latter structure as a site of a robust sensory template. Moreover, the earlier findings in NCM are closely paralleled by our observation that in birds in whom the BOLD response to TUT has not saturated, namely repeaters, BOLD activation by TUT show a slight trend towards positive linear correlation with how well the pupils imitate the tutor's song. The trend becomes statistically significant when tutor-pupil song motif comparison are done using the symmetric as opposed to asymmetric time course method in Sound Analysis Pro [26]. Since this method preserves the sequence of syllables in the comparison, our result might suggest that copying of the temporal frame from song is better correlated with the sensory representation of the tutor's song rather than copying of its spectral content. This interpretation is consistent with our earlier finding of dissociation between frame and content in tutor's song imitation in repeaters [4].

Furthermore, these data suggest that reduced response strength in adults might be related to the alterations of the song syntax despite there being no significant deficiency in the learning of tutor's song motifs. The latter point can be inferred from the fact that there is no significant difference in mean measures of tutor-pupil song motif similarity between repeaters and non-repeaters. The finding that neurotoxic lesions of NCM in adults affect song perception but not production [38] does not contradict our results. It conforms to the notion that long persistence of the tutor's song memory might not be required for the maintenance of BOS, once learned.

The enhancement of the BOLD response to the unfamiliar CON stimulus in repeaters might suggest changes in a related or shared mechanism of fine tuning of the gain of sensory activation based on stimulus familiarity. At the synaptic level, this might reflect plasticity of excitatory or inhibitory transmission. It has been shown recently that nearly half of the neurons in NCM are inhibitory in nature, and that they play an important role in auditory processing in this structure [39]. So quite apart from possible changes in excitatory transmission, if the reduced response to a highly familiar song such as that of the tutor turns out to be due to long-term neuroplastic upregulation of synaptic inhibition, then an unfamiliar stimulus might produce a stronger response because it occurs in the presence of reduced amounts of inhibition. A difference in control and plasticity of synaptic inhibition might then be responsible for the differences in responsiveness to TUT and CON between repeaters and non-repeaters. The facts that the BOLD hemodynamic response mirrors the directionality of excitatory and inhibitory post-synaptic potentials rather than spiking activity of neurons, and their opposing effects on each other, are now well supported [40], [41], [42].

To conclude, the findings of this paper provide the first evidence for a neural correlate of song representation in birds that have a tendency to produce learned songs with aberrant or variant song syntax involving variable repetitions of sub-motif elements. A differential change in the hemodynamic response measured by a non-invasive method to a developmentally significant stimulus and a novel stimulus, in songbirds that produce variant song output, is a new finding significant from a comparative standpoint. Our result opens up a new approach to studying the neurobiology of songbird models of vocal disturbances, longitudinally over the course of development, using a technique that is non-invasive and is repeatedly applied in the same animal. Future fMRI studies designed to track the development of altered BOLD responsiveness to tutor's song, as well as other conspecific songs that vary in familiarity and saliency, would be able to test the extent to which establishment of the sensory song template and/or long-term maintenance of sensory song memories, contribute to the development of normal vocal motor output and syntax of bird's own song.

These studies might open new possibilities for the utilization of the songbird model to explore the neural basis of human dysfluencies. The anatomical areas that show activation in response to song stimuli in the zebra finch have parallels in the human brain. Field L could be regarded as analogous to Heschl's gyrus. NCM and CM have similarities with the auditory association areas. A few observations in people who stutter that might have some relationship with our findings are as follows. First, even though most stutterers repeat the first syllable of words, in several cases of stuttering due to neurological deficits, repetitions occur on the final syllable of words, in analogy with the repetitions that we observe in zebra finches [43], [44], [45], [46], [47]. As far as auditory processing in stutterers is concerned, alterations have been implicated in a number of recent studies, both involving electrophysiological recordings [48] and functional brain scanning [49]. Stutterers have increased activation in the left middle and superior temporal gyri and right insula, primary motor cortex and supplementary motor cortex during the passive listening condition, relative to non-stutterers [49]. During speech production the left Heschl's gyrus shows reduced activation in stutterers compared to controls [50]. Increased bilateral activations in Heschl's gyri have also been shown during speech and non-speech production tasks, and reduced activations in auditory areas in speech and non-speech perceptual tasks [51]. A meta-analysis of imaging studies also shows reduced activations of auditory areas to hearing of one's own speech in stutterers [52]. Auditory processing deficits, as measured by activity in the right superior and middle temporal gyri, are also implicated in positron emission tomographic studies [53]. It would therefore be worthwhile to explore in future studies, how closely fMRI correlates of stuttered vocal output in zebra finches can model those in people who stutter, and thereby derive insights into the neurodevelopmental basis of stuttering.
